# Exploring caregiver burden and coping responses in severe mental illness in rural India

**DOI:** 10.6026/973206300220397

**Published:** 2026-01-31

**Authors:** Murali M.R, Shashank K.J, Anuradha V, Heena Dixit Tiwari, Abhimanyu Singh, Rahul Tiwari, Afroz Kalmee Syed

**Affiliations:** 1 Department of Psychiatry, JSS Academy of Higher Education & Research, Mysore, Karnataka, India; 2 Department of Community Medicine, Chikkamagaluru Institute of Medical Sciences, Chikkamagaluru, Karnataka, India; 3 Department of Business Management, UCCBM Mahatma Gandhi University Nalgonda, Telangana, India; 4 Blood Cell, Commissionerate of Health and Family Welfare, Government of Telangana, Hyderabad, India; 5 Department of Pediatric and Preventive Dentistry, BBD College of Dental Sciences, Lucknow, Uttar Pradesh, India; 6 Department of Dental Research Cell, Dr. D. Y. PatiVidyapeethollege & Hospital, Dr. D. Y. Patil Vidyapeeth (Deemed to be University), Pimpri, Pune, India; 7 Department of Oral and Maxillofacial Pathology, Writing and Publications, Tenali, Andhra Pradesh, India

**Keywords:** Caregivers, mental disorders, rural health, adaptation, psychological, qualitative research

## Abstract

Caregiver burden among families supporting individuals with severe mental illness (SMI) in rural India remains inadequately explored despite its critical influence on treatment adherence and family well-being. Severe mental illness imposes substantial psychological, social and economic strain on caregivers living in resource-limited rural settings. Therefore, it is of interest to explore the lived experiences, perceived burden and coping responses among primary caregivers of individuals with SMI in rural India. A qualitative descriptive design was adopted and in-depth interviews were conducted with caregivers recruited from three rural primary health-care catchment areas. Thematic analysis revealed multidimensional caregiver burden characterized by emotional exhaustion, financial hardship, disrupted family routines, social isolation and stigma. Coping responses ranged from problem-focused strategies such as information-seeking and structured caregiving routines to emotion-focused and faith-based practices. Participants consistently reported unmet needs for psychoeducation, accessible mental-health services and respite support. Thus, we show the necessity of integrating caregiver-centred interventions, community-based psychoeducation and strengthened rural mental-health outreach services to reduce caregiver burden and improve continuity of care for individuals with SMI.

## Background:

Caregivers of individuals living with severe mental illness (SMI)-including schizophrenia, bipolar disorder and chronic psychotic disorders-serve as the primary source of emotional, social and economic support in low-resource rural settings. The caregiving role often extends beyond supervision to include medication management, behavioural monitoring and coordination with overstretched health-care systems. Globally, caregiver burden has been shown to encompass psychological distress, financial strain, social isolation, strained family relationships and long caregiving hours [1]. Recent studies from low- and middle-income countries highlight that rural caregivers face elevated levels of stress due to limited service availability, poor mental-health literacy, stigma and treatment delays [2, 3]. Additionally, caregiving experiences are shaped by cultural expectations that normalize family responsibility, creating situations in which emotional strain is underreported or unaddressed [4]. Coping responses among caregivers may range from adaptive strategies-such as seeking information and community support-to emotion-focused or avoidance-based approaches, including faith- healing reliance and withdrawal [5, 6]. Despite increasing attention to mental-health disparities within India, research specifically examining caregiver burden and coping responses in rural SMI contexts remains limited. Therefore, it is of interest to understand their lived experiences to inform supportive interventions and strengthen community-based psychiatric care.

## Materials and Methods:

A descriptive qualitative design was employed to explore caregiver burden and coping responses among caregivers of individuals with SMI in rural India. The study was conducted between January and June 2025 across three rural primary-health catchment areas located 40-80 km from the nearest district hospital. Adults aged ≥18 years who were the primary caregivers of individuals diagnosed with schizophrenia, bipolar affective disorder with psychosis, or schizoaffective disorder for at least one year were eligible. Caregivers with acute medical illness or cognitive impairment were excluded. Purposive sampling was used to recruit 20 participants through community health workers, outpatient psychiatry units and district mental-health outreach clinics. Semi-structured interviews were conducted in the local language at participants' homes or community centers. Interview guides explored caregiving routines, emotional and financial strain, social relationships, stigma experiences, coping responses and perceived support needs. Interviews lasted 45-60 minutes, were audio- recorded with consent, transcribed verbatim and translated into English. Data were analyzed using inductive thematic analysis. Two researchers independently coded transcripts, identified meaning units and grouped them into preliminary categories. Themes were finalized through iterative comparison and consensus. Trustworthiness was ensured through reflexive memoing, peer debriefing and verification of emerging findings with a subset of participants. Ethical approval was obtained from an institutional ethics committee and written informed consent was secured from all participants. Confidentiality and anonymity were strictly maintained.

## Results:

Table 1 shows that caregivers were predominantly middle-aged (mean age 44.6 years) and primarily female (65%), reflecting gendered caregiving expectations in rural households. Parents and spouses constituted most caregivers. Financial vulnerability was common, with 70% reporting monthly household income below INR 10,000. Notably, 80% lived more than 30 km from mental-health services, underscoring the geographic disadvantage and logistical challenges associated with accessing psychiatric care for chronic SMI management. Table 2 summarizes five major thematic domains. Caregivers described significant emotional, financial and social burden. Stigma emerged as a pervasive community influence shaping daily interactions and care-seeking decisions. Coping responses varied: some caregivers used problem-focused strategies such as structured routines and information-seeking, while others relied on faith-based practices or emotional withdrawal. Across interviews, participants consistently identified unmet needs, particularly practical guidance, accessible services and respite support.

## Discussion:

This study provides an in-depth understanding of caregiver burden and coping responses among families supporting individuals with SMI in rural India. Findings demonstrate that caregiving is shaped by intersecting emotional, social and economic pressures, consistent with global and regional evidence. Emotional strain reported by participants aligns with recent studies documenting high psychological distress and depressive symptoms among caregivers of chronic mental illness [7]. Financial burden was prominent in this study, reflecting broader patterns seen in rural low-resource settings, where caregivers bear substantial out-of- pocket costs for transport, medication and lost wages [8]. Stigma emerged as a marked influence on caregiver experiences. Consistent with work by Rapiya *et al.* (2025), caregivers described blame, social distancing and concealment of illness as ongoing challenges that limited support networks and reinforced isolation [9]. Local cultural beliefs and misunderstanding of mental illness further contributed to caregiving strain, echoing findings from Ghana, Ethiopia and other LMICs where caregivers negotiate community judgment and cultural attributions of illness [10, 11]. Coping responses varied widely. Adaptive coping- such as seeking information, maintaining structured routines and family collaboration-mirrors findings from cross-sectional studies identifying problem- focused coping as a protective factor against burden [12]. Faith-based coping was prevalent among participants, aligning with the qualitative work of Daliri *et al.* (2024), who reported reliance on prayer and spiritual interpretation as common coping pathways in rural African contexts [13]. While faith practices may offer emotional relief, they may also delay biomedical care if relied upon exclusively. Unmet support needs identified in this study reinforce prior conclusions that rural caregivers require structured psychoeducation, accessible outreach services and sustained contact with mental-health professionals [14]. Studies from rural India and Ethiopia similarly emphasize gaps in health-worker engagement, medication continuity and caregiver-focused interventions [15]. These findings highlight the potential value of community health-worker-led psychoeducation programs, telepsychiatry support and caregiver support groups, which have demonstrated positive outcomes in several LMIC contexts. Overall, this study underscores the urgent need for integrated caregiver-centered approaches within rural mental-health systems. Strengthening continuity of care through community outreach, reducing stigma through targeted community awareness and supporting caregivers through structured interventions may substantially reduce long-term burden and improve outcomes for both caregivers and individuals with SMI.

## Conclusion: 

Caregivers supporting individuals with SMI in rural India experience substantial emotional, financial and social burden, with coping responses shaped by cultural, familial and contextual factors. Strengthening rural mental-health systems through psychoeducation, community engagement and caregiver-focused support interventions is essential to enhance caregiver resilience and continuity of care.

## Figures and Tables

**Table 1:** Socio-demographic characteristics of caregivers (n=20)

**Variable**	**Category**	**Frequency (%)**
Age (years)	Mean ± SD	44.6 ± 10.8
Gender	Female	13 (65%)
	Male	7 (35%)
Relationship to patient	Parent	9 (45%)
	Spouse	6 (30%)
	Sibling/Adult child	5 (25%)
Monthly household income	< INR 10,000	14 (70%)
Distance to nearest mental-health facility	>30 km	16 (80%)

**Table 2:** Socio-demographic characteristics of caregivers (n=20)

**Major Theme**	**Subthemes Identified**
1. Multidimensional Caregiver Burden	Emotional strain, financial hardship, disrupted family routines, social isolation
2. Stigma and Community Dynamics	Blame, labeling, avoidance by neighbors, concealment of illness
3. Adaptive Coping Responses	Seeking information, family collaboration, structured routines
4. Faith-Based and Emotion-Focused Coping	Prayer, temple visits, reliance on spiritual healers
5. Unmet Support Needs	Need for psychoeducation, respite, outreach services, medication continuity
